# Prevalence and Transfusion Risks of Occult Hepatitis B Infection Among HBcAb-Positive Blood Donors in a High-Endemic Region

**DOI:** 10.3390/diagnostics15040486

**Published:** 2025-02-17

**Authors:** Ahmed Subeh Alshrari, Shuaibu Abdullahi Hudu, Sa‘adatu Haruna Shinkafi, Albashir Tahir, Halima Yunusa Raji, Abdulgafar Olayiwola Jimoh

**Affiliations:** 1 Medical Laboratory Technology Department, Faculty of Applied Medical Science, Northern Border University, Arar 91431, Saudi Arabia; 2 Department of Basic and Clinical Medical Sciences, Faculty of Dentistry, Zarqa University, Zarqa 13110, Jordan; 3 Department of Medical Microbiology and Parasitology, Faculty of Basic Clinical Sciences, College of Health Sciences, Usmanu Danfodiyo University, Sokoto 840232, Nigeria; 4 Department of Microbiology and Parasitology, Usmanu Danfodiyo University Teaching Hospital, Sokoto 840001, Nigeria; saadat.haruna@gmail.com (S.H.S.); limaraj2000@gmail.com (H.Y.R.); 5 Department of Pharmacology, Faculty of Basic Medical Sciences, Sa’adu Zungur University, Bauchi 751105, Nigeria; albashirtahir@gmail.com; 6 Department of Pharmacology and Therapeutics, Faculty of Basic Clinical Sciences, College of Health Sciences, Usmanu Danfodiyo University, Sokoto 840001, Nigeria; jimoh.abdulgafar@udusok.edu.ng

**Keywords:** hepatitis B virus, hepatitis B, occult hepatitis B infection, HBcAb, HBsAg

## Abstract

**Background:** Hepatitis B virus (HBV) remains a significant global health concern, particularly in sub-Saharan Africa, where endemicity is high. Occult hepatitis B infection (OBI) presents a unique challenge to transfusion safety, as HBV DNA may persist in HBsAg-negative individuals. This study examines the prevalence of HBcAb positivity among blood donors at Usmanu Danfodiyo University Teaching Hospital (UDUTH), Sokoto, and assesses the risk of HBV transmission. **Methods:** A cross-sectional study was conducted among 200 blood donors. Samples were screened for HBsAg and HBV serological markers using a rapid assay and ELISA. HBcAb-positive samples were analyzed for HBV DNA using real-time polymerase chain reaction (qRT-PCR). Viral loads were quantified, and socio-demographic characteristics were recorded. **Results:** HBcAb was detected in 57 (28.5%) of the 200 donors. The most common serological pattern among donors was HBsAg-negative and HBcAb-negative (69%). Among these HBcAb-positive donors, HBV DNA was detected in three cases (1.5%), with viral loads of 753.1, 2.193 × 10^4^, and 4.538 × 10^4^ IU/mL. The presence of HBV DNA in these donors confirms the risk of OBI transmission through transfusion. Socio-demographic analysis revealed that 48.5% of donors were aged 26–35 years, 23.5% were aged 18–25 years, 23% were aged 36–42 years, and 2.5% were either 43–50 or above 50 years of age, of which 99.5% were male. These findings highlight a significant prevalence of HBcAb positivity and OBI, aligning with studies in similar high-endemic settings. **Conclusions:** HBcAb positivity and OBI represent significant transfusion risks in endemic regions. The presence of HBV DNA in 1.5% of HBcAb-positive donors in the study population highlights the limitations of HBsAg-based screening. Incorporating nucleic acid testing (NAT) into routine blood donor screening protocols is critical to enhancing transfusion safety. Further research is needed to evaluate the feasibility and cost-effectiveness of such interventions in resource-limited settings.

## 1. Introduction

Hepatitis B virus (HBV) infection remains a noteworthy challenge to public health efforts, particularly in regions such as sub-Saharan Africa, where the endemicity is high. According to the World Health Organization (WHO), about 296 million people live with chronic HBV infection, leading to chronic liver complications and increased death rates [1,2]. The prevalence of HBV infection in Nigeria is especially alarming, with prevalence rates ranging between 8 and 15% across different population groups. This high burden is compounded by the complexities surrounding blood donation practices, where gaps in effective donor screening systems pose significant risks for transmission [3].

Among the various serological markers related to HBV infection, antibodies to the hepatitis B core antigen (HBcAb) have garnered particular attention due to their role in identifying previous exposure to the virus [4]. Notably, individuals who tested negative for hepatitis B surface antigen (HBsAg), the conventional marker for blood screening, may harbor occult HBV infection (OBI). This condition is characterized by the presence of replication-competent HBV DNA in the liver, in the presence or absence of HBV DNA in the blood of individuals testing negative for HBsAg, which poses a silent yet significant threat to transfusion safety [5,6]. The condition carries silent but serious risks, especially in situations like blood transfusions and organ transplantation, where undetected HBV can have serious consequences for recipients. Antibodies to hepatitis B core antigen (HBcAb) are important clues for prior HBV interactions but are notoriously difficult to interpret. In many resource-limited countries like Nigeria, blood donor screening for HBV primarily relies on HBsAg testing due to its affordability and practicality. However, this approach does not detect OBI, which underscores the need for more sensitive diagnostic tools. Investigation of the low prevalence of HBcAb positivity and its possible implications for OBI typically involves both serological and molecular analyses. OBI is confirmed by downstream confirmatory testing such as indirectly detecting HBV DNA by nucleic acid testing (NAT) or polymerase chain reaction (PCR) [7,8].

Studies indicate that blood from HBsAg-negative, HBcAb-positive donors can still transmit HBV at rates of 2 to 8.6%, with transmission risks influenced by viral load, component volume, and recipient immune status [9]. Many studies have established the high rate of OBI among HBsAg-negative, HBV-positive cases. In high-endemic settings, the prevalence of OBI in blood donors can be 1% to 20%, depending on the population and diagnostic methods [10]. In another study in Nigeria, the reported OBI prevalence rate was around 6%, suggesting a potential risk of transfusion-transmitted infection in that region [11]. The primary diagnostic challenge lies in the inability of serology alone to diagnose OBI, necessitating the use of molecular techniques for confirmation. Although HBcAb positivity indicates past HBV exposure, individually, it may represent resolved infection, OBI, or a false-positive result because of inherent limitations in the assay [12]. Polymerase chain reaction-based assays that can detect low levels of HBV DNA are now considered the gold standard for diagnosis [7]. However, their expensive nature and complexity restrict their widespread deployment in resource-limited environments. HBV and its occult form present major public health problems, especially in regions with high endemicity such as sub-Saharan Africa.

The risk of transfusion-related HBV transmission from HBcAb-positive donors is highlighted by research showing that OBI transmission occurs at an estimated rate of 3.9 to 6.5 cases per million donations, a figure substantially higher than the residual risk during the HBV window period [13]. While some studies argue that blood from HBcAb-positive donors may be relatively safe due to low viral loads or neutralizing antibodies, growing evidence points to a quantifiable risk that cannot be ignored [9,13,14]. These findings emphasize the need for advanced screening protocols to prevent such occurrences. Furthermore, the interpretation of HBcAb positivity is particularly challenging in vaccinated populations. Finding solutions to the dangers of OBI demands a multi-pronged response combining novel diagnostic tests, facilitating policy changes, and bolstering public health initiatives. While universal HBV vaccination is critical for significantly lowering the global HBV burden, screening for OBI is an important measure for ensuring transfusion safety. Usmanu Danfodiyo University Teaching Hospital (UDUTH) in Sokoto, Nigeria serves a diverse patient population and plays a critical role in providing blood transfusion services. However, existing donor screening protocols, which rely predominantly on HBsAg testing, may fail to adequately address the risks associated with HBcAb positivity and OBI. This study seeks to evaluate the presence of HBcAb positivity among blood donors and its implication for transfusion safety.

## 2. Methodology

### 2.1. Ethical Considerations

Approval for the study was obtained from Usmanu Danfodiyo University Teaching Hospital, Sokoto Health Research Ethics Committee Reference Number: UDUTH/HREC/2023/630/2). and informed consent was obtained from all participants, ensuring voluntary participation. Confidentiality was strictly maintained by anonymizing all samples and data, which were accessible only to authorized personnel only.

### 2.2. Study Location and Participants

The study was conducted at the Blood Bank of the Hematology Department, UDUTH, located in Sokoto, Nigeria. A total of 200 voluntary blood donors were recruited, representing diverse socio-demographic backgrounds. The eligibility criteria included healthy donors aged 18–50 years, free from chronic diseases, who were willing to provide informed consent. Donors with HBsAg positivity or known HBV-related conditions were excluded.

### 2.3. Sample Size

The minimum sample size for the study was determined using the sample size formula for proportions [15]:

$$n=\frac{Z^{2}\times p\times (1-p)}{E^{2}}$$

where *Z* is the Z-score corresponding to the desired confidence level (a 95% confidence level corresponds to *Z* ≈ 1.96); *p* is the estimated prevalence of HBcAb positivity (5.3%), as indicated by a previous study conducted in the same setting [16]; and *E* is the margin of error (set at 5% or 0.05).

This calculation yielded a minimum sample size of 77 for the study. However, to increase statistical robustness, the final study population consisted of 200 blood donors.

### 2.4. Specimen Collection and Storage

About 4–5 mL of venous blood was collected from each donor using sterile ethylenediaminetetraacetic acid (EDTA) bottles labeled with the donor’s identity number and stored at room temperature for two hours. The serum was separated by centrifugation at 8000 rpm for 5 min and aliquoted into plastic vials for subsequent analysis. The specimens were stored at −25 °C to −15 °C until use.

### 2.5. Qualitative HBV Test

The Hightop^®^ HBV 5-in-1 HBV Marker Rapid Test Panel (Qingdao Hightop Biotech Co. Ltd., Qingdao, China) was employed for the qualitative detection of HBV serological markers, including Hepatitis B Surface Antigen (HBsAg), Hepatitis B Surface Antibody (HBsAb), Hepatitis B Envelope Antigen (HBeAg), Hepatitis B Envelope Antibody (HBeAb), and Hepatitis B Core Antibody (HBcAb). The procedure was carried out according to the manufacturer’s instructions. Before running the test, the kit components were allowed to equilibrate at room temperature for approximately 30 min to ensure optimal performance. Three drops (80–100 μL) of serum were carefully dispensed vertically into the designated sample well of the test panel. The panel was then monitored for a reaction, and the results were interpreted within 15–20 min (Table 1).

### 2.6. DNA Extraction

The viral genomic DNA was extracted directly from the donor’s serum using the Bioer nucleic extraction reagent (Hangzhou Bioer Technology Co., Ltd., Hangzhou, China) according to the manufacturer’s instructions. Briefly, 100 µL of the serum specimen was processed with 100 µL of Solution A and centrifuged at 1300 rpm for 10 min. The resulting precipitate was resuspended in 25 µL of Solution B and incubated at 100 °C for 10 min. The supernatant was extracted after conjugation at 13,000 rpm for 10 min. The controls (negative, strong positive, and critical positive) were extracted as the unknown. The Negative Control was used to rule out contamination during the DNA extraction and amplification process, ensuring that any detected signal solely originated from the test samples. The Strong Positive Control validated the sensitivity of the extraction process, confirming that the method was capable of detecting even low concentrations of HBV DNA, and the Critical Positive Control served to confirm reliable detection and consistency of the results, ensuring the accuracy of the assay under study conditions. The resulting DNA extract was used directly for HBV viral DNA quantification or stored at −20 °C until use.

### 2.7. Real-Time PCR Quantification for HBV DNA

The quantitative reaction was carried out using a commercial HBV PCR fluorescence quantitative detection kit (Hangzhou Bioer Technology, Hangzhou, China). The kit combines the technologies of nucleic acid amplification and a hybridization probe to detect HBV DNA. The reagents contain Taq polymerase for template DNA synthesis and UDG for prevention of PCR contamination. PCR thermal cycling was performed in a Magnetic Induction real-time PCR cycler (Biomolecular Systems, Upper Coomera, Australia). The reaction conditions were 37 °C for 5 min, followed by 94 °C for 5 s and 60 °C for 40 s (Table 2). A series of four standards (1–5 × 10^7^ IU/mL, 1–5 × 10^6^ IU/mL, 1–5 × 10^5^ IU/mL, 1–5 × 10^4^ IU/mL) was included to create standard curves for quantitative analysis of specimen HBV DNA concentration. The internal amplification control was used to monitor the presence or absence of PCR inhibition.

Occult HBV infection was defined as the presence of HBV DNA with serological results that were HBsAg-negative and anti-HBc-positive.

### 2.8. Data Analysis

Descriptive statistics were used to summarize demographic characteristics and serological profiles using statistical software (IBM SPSS, version 25).

## 3. Results

### 3.1. Socio-Demographic Characteristics of Blood Donors

The socio-demographic characteristics of the 200 participants included in the study (Table 3) reveal a relatively young cohort, with most donors falling into the 26–35 age group. This age group, comprising 97 individuals (48.5%), represents nearly half of the total participants. The second most prominent group is the 18–25 age range, with 47 participants (23.5%). Together, these two age groups account for 72% of the study population. The 36–42 age group includes 46 participants (23.0%), and 5 participants (2.5%) are aged 43–50 years or above 50 years of age (5 individuals, 2.5%). The gender distribution of the participants shows a marked disparity, with nearly all donors being male (199 individuals, 99.5%). Only 1 female donor (0.5%) is included in the study.

The distribution of hepatitis B serological markers among the study participants is summarized in Table 4. The most common serological pattern observed was HBsAg-negative, HBsAb-negative, HBeAg-negative, HBeAb-negative, and HBcAb-negative. This pattern, found in 138 participants (69.0%), indicates that the majority of donors had no evidence of past or current hepatitis B virus (HBV) infection or immunity. A small subset of participants (one donor, 0.5%) displayed the following serological profile: HBsAg-negative, HBsAb-positive, HBeAg-negative, HBeAb-negative, and HBcAb-negative. This pattern signifies immunity acquired through vaccination.

A significant proportion of participants tested positive for the hepatitis B core antibody (HBcAb), indicating past exposure to HBV. Among these, 19 participants (9.5%) exhibited the following pattern: HBeAg-negative, HBeAb-positive, and HBcAb-positive. This was indicative of resolved infections with a protective immune response. Another 40 participants (20.0%) were HBeAg-negative, HBeAb-negative, and HBcAb-positive, a profile often associated with occult HBV infection or past exposure without clear immune resolution. Finally, two participants (1.0%) displayed a profile that was HBeAg-positive, HBeAb-negative, and HBcAb-positive, indicating active viral replication and higher infectivity.

### 3.2. Detection of HBV DNA

**Figure 1:**
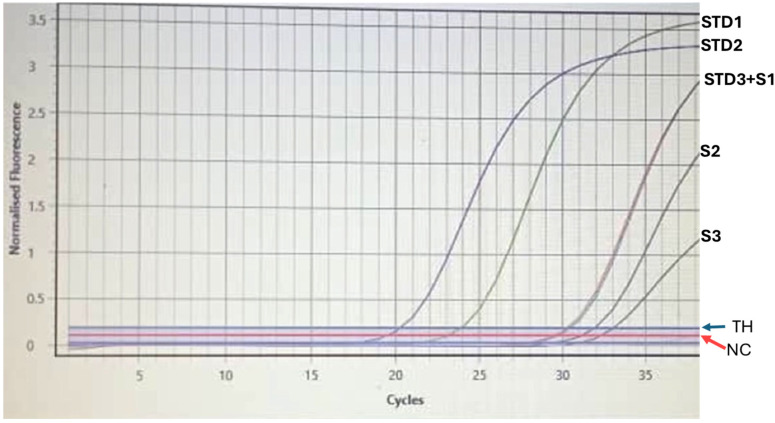
HBV DNA levels detected in three HBsAg-negative, anti-HBc-positive donors. STD: standard; S1–S3: donor’s samples; TH: threshold; NC: Negative Control; STD3+S1: donor’s sample overlapping with standard.

Among the 61 participants who tested HBsAg-negative and anti-HBc-positive in the qualitative HBV test, HBV DNA was detected in 3 individuals. This represents 7.5% of the anti-HBc-positive donors, highlighting the presence of occult HBV infection in this subgroup. The detected HBV DNA levels varied significantly among the three donors, with measured values of 753.1 IU/mL, 2.193 × 104 IU/mL, and 4.538 × 104 IU/mL, respectively (Figure 1). The two participants that displayed HBeAg-positive, HBeAb-negative, and HBcAb-positive profiles, indicating active viral replication and higher infectivity, are among the three with detectable HBV DNA, while one had an HBeAg-negative, HBeAb-negative, and HBcAb-positive pattern.

## 4. Discussion

The findings of this study provide critical insights into the risks associated with the use of HBcAb-positive blood donors for transfusion services, a subject of growing concern in blood safety research. Among the 200 donors evaluated, 61 (30.5%) tested positive for HBcAb, despite being negative for HBsAg. This aligns with earlier studies from high-HBV-endemic regions, including sub-Saharan Africa, which report similar HBcAb positivity rates among blood donors [17]. The presence of HBcAb indicates prior HBV exposure and raises the potential for OBI, as HBV DNA may persist in such individuals despite the absence of detectable HBsAg. OBI poses significant challenges to transfusion safety, as even low viral loads can be sufficient to cause infection in immunocompromised individuals, neonates, or those receiving multiple transfusions [5]. Moreover, the high prevalence of HBcAb positivity reflects the widespread exposure to HBV in the general population, emphasizing the need for improved screening protocols and preventive measures.

The demographic analysis revealed notable age and gender disparities. Most donors were aged 26–35 years (48.5%), followed by those aged 18–25 years (23.5%), reflecting that a younger demographic is more likely to donate blood. These findings align with other studies conducted in sub-Saharan Africa, where younger and middle-aged males are the dominant blood donors due to cultural and societal factors [18,19]. The gender distribution was strikingly skewed, with 99.5% of donors being male, reflecting cultural norms, societal expectations, and misconceptions about blood donation among women. This imbalance may limit the diversity of blood supplies and exacerbate shortages during emergencies. Addressing these issues requires implementing targeted educational campaigns, addressing misconceptions, and providing incentives such as complementary checks to encourage female participation [20]. Directed donation, a practice where a donor or their family specifies who will receive a blood donation, allows individuals to choose recipients and fosters a personal connection to the act of giving. However, this practice has raised concerns and has been banned or restricted in many regions due to various ethical and safety issues including coercion and pressure, infectious disease risk, and equity, limiting its use to specific patient populations, such as those with rare blood types or immunoglobulin A deficiencies [21,22].

The serological profiles of donors further highlighted the complexity of HBV exposure and immunity. The most common serological pattern was HBsAg-negative, HBsAb-negative, HBeAg-negative, HBeAb-negative, and HBcAb-negative; this pattern was observed in 69% of donors. This pattern suggests a naïve status with no prior exposure to HBV. However, 20% of donors were HBeAg-negative, HBeAb-negative, and HBcAb-positive, and 9.5% were HBeAg-negative, HBeAb-positive, and HBcAb-positive. Only 1% were HBeAg-positive and HBcAb-positive. This heterogeneity in serological patterns is consistent with findings from other studies such as those by Fasola et al. (2022), underscoring the complex interplay between HBV infection, immune response, and potential reactivation risks [17].

Among the HBcAb-positive, HBsAg-negative donors, HBV DNA was detected in three individuals (1.5%) with viral loads of 753.1, 2.193 × 10^4^, and 4.538 × 10^4^ IU/mL. While the proportion is small, it represents a significant transfusion risk, particularly for immunocompromised recipients. The prevalence of OBI in this study aligns with findings from other HBV-endemic regions where OBI prevalence varies widely, ranging from 0.5% in Ghana [23] to 17% in other Nigerian studies [24]. Such variability can be attributed to several factors, including population-related factors, methodological variations in diagnostic techniques, and assay sensitivity.

The detection of HBV DNA in HBcAb-positive donors demonstrates the inadequacy of relying solely on HBsAg testing. Conventional serological testing fails to identify OBIs, leaving blood transfusion recipients vulnerable to undiagnosed HBV infections. Nucleic acid testing (NAT), which can detect low levels of viral DNA, has emerged as a critical tool for improving transfusion safety in HBV-endemic regions [5]. However, the implementation of NAT in resource-limited settings faces significant barriers, including high costs, lack of infrastructure, and limited skilled personnel. Implementing affordable HBV DNA testing in all blood banks in Nigeria would require a phased approach to NAT, starting with regional or referral blood banks, allowing for a scalable and cost-effective rollout. The key to this process would be addressing financial barriers through collaborative funding initiatives involving international health organizations, public–private partnerships, and successful models like the Global Fund and Gavi. Training healthcare personnel and improving laboratory infrastructure are also critical for effective NAT integration [25].

To improve blood transfusion safety in remote and underserved areas, point-of-care (POC) testing for HBcAb and HBsAg should be prioritized as part of routine donor screening. This requires governmental support systems to provide funding, deploy portable diagnostic kits, and train healthcare personnel to ensure effective implementation and sustainability. In areas or systems where NAT cannot be implemented immediately, the addition of anti-HBc serological testing could serve as an intermediate solution if HBV testing were extended beyond HBsAg to include anti-HBc markers. The application of anti-HBc screening in conjunction with confirmatory testing has proved effective in improving the detection of OBI and lowering its related risks. However, isolated anti-HBc positivity, particularly in high-endemic populations, usually represents resolved infections rather than active OBI [26]. This underscores the need for strong confirmatory procedures that reduce the risk of inappropriate exclusion from donation while also safeguarding transfusion safety. Moreover, isolated anti-HBc positivity with diagnostic ambiguities, especially when the patient has been vaccinated against HBV, requires the design of confirmatory strategies specific to anti-HBc, such as assessment of HBV DNA, to overcome the limitations of serological assays and an appropriate balance of safety vs. adequate blood supply [27].

The findings highlight the pressing need to re-evaluate donor screening protocols. A mixed method combining phased NAT implementation and enhanced serological testing can address limitations in current practices. Capacity-building initiatives, including investments in infrastructure and training, are essential to ensure sustainable improvements. Local governments, international donors, and other stakeholders must collaborate to coordinate efforts and resources. A systematic and integrated approach will improve transfusion safety and reduce the burden of transfusion-transmitted HBV in high-endemic areas, aligning with WHO guidance for universal health coverage in blood safety [28]. In addition, the high prevalence of HBcAb positivity highlights the importance of preventive measures, including vaccination campaigns and public health education. For health systems, managing HBcAb-positive donors presents ethical and logistical challenges, particularly when it comes to balancing donor deferral with maintaining adequate blood supplies.

Future initiatives should prioritize ensuring comprehensive vaccination coverage against HBV in Nigeria, particularly given the concerning data that only one blood donor exhibited a serological pattern consistent with vaccination. While exploring the genetic and environmental factors influencing HBV persistence and transmission is valuable, a more straightforward and effective approach would be to implement robust birth dose and catch-up vaccination programs for infants, children, and adults. This strategy would directly address the low vaccination rates contributing to HBV endemicity and the associated risks of OBI in blood donors. Such studies could significantly enhance transfusion safety and reduce the prevalence of HBV in the population, aligning with international health standards and improving overall public health outcomes.

## 5. Conclusions

This study highlights the considerable risks associated with using HBcAb-positive donors for blood transfusion, particularly in regions with high HBV endemicity. The prevalence of HBcAb positivity (28.5%) and the detection of HBV DNA in 1.5% of these donors emphasize the limitations of current HBsAg-based screening protocols when it comes to identifying OBI. These findings provide compelling evidence for the urgent need to enhance blood transfusion safety strategies through the adoption of advanced diagnostic tools and protocols tailored to the realities of resource-limited settings.

To reduce the risks of OBIs, integrating NAT into routine donor screening is crucial, as it complements serological methods and detects HBV DNA in HBsAg-negative donors. Expanding screening to include anti-HBc testing can refine donor eligibility. However, these efforts require investments in laboratory infrastructure, supply chains, and personnel training. Additionally, addressing gender disparities in blood donation through culturally sensitive campaigns and incentives for female donors is essential to ensure a resilient blood supply. Public awareness campaigns should also encourage safe donation practices. Policy interventions are key to sustaining these improvements; thus, governments should work with international health organizations to establish national HBV screening guidelines, with sufficient funding and oversight. Future research should examine the long-term effects of enhanced screening protocols and explore cost-effective approaches for resource-constrained settings.

## Figures and Tables

**Table 1:** Classical interpretation of HBV markers.

Marker	Indication
HBsAg	Active HBV infection
HBsAb	Immunity (post-infection or vaccination)
HBeAg	High-infectivity phase
HBeAb	Transition to lower infectivity
HBcAb	Previous exposure to HBV

**Table 2:** PCR Conditions.

Step	Temperature	Time
UDG Activation	37 °C	5 min
Denaturation	94 °C	5 s
Amplification (40 cycles)	95 °C/60 °C	40 s

**Table 3:** Participants’ socio-demographic characteristics.

Age in Years	Frequency	Percent
18–25	47	23.5
26–35	97	48.5
36–42	46	23.0
43–50	5	2.5
Above 50	5	2.5
**Gender**		
Male	199	99.5
Female	1	0.5

**Table 4:** Distribution of hepatitis B serological markers in the sample population.

HBsAg	Anti-HBs Ab	HBeAg	Anti-HBeAb	Anti-HBcAb	Frequency	Percentage
-	-	-	-	-	138	69.0
-	+	-	-	-	1	0.5
-	-	-	+	+	19	9.5
-	-	-	-	+	40	20
-	-	+	-	+	2	1

Note: Negative reaction (-) and Positive reaction (+).

## Data Availability

The authors will make the raw data supporting this article’s conclusions available upon request.

## Abbreviations

The following abbreviations are used in this manuscript:

HBV	Hepatitis B Virus.
HBcAb	Hepatitis B Core Antibody.
HBsAg	Hepatitis B Surface Antigen.
OBI	Occult Hepatitis B Infection.
ELISA	Enzyme-Linked Immunosorbent Assay.
qRT-PCR	Quantitative Real-Time Polymerase Chain Reaction.
NAT	Nucleic Acid Testing.
Ct	Cycle Threshold.
EDTA	Ethylenediaminetetraacetic Acid.
DNA	Deoxyribonucleic Acid.
HBeAg	Hepatitis B Envelope Antigen.
HBeAb	Hepatitis B Envelope Antibody.
HBsAb	Hepatitis B Surface Antibody.
